# Cleavage of non-polar C(*sp*^2^)‒C(*sp*^2^) bonds in cycloparaphenylenes via electric field-catalyzed electrophilic aromatic substitution

**DOI:** 10.1038/s41467-022-35686-4

**Published:** 2023-01-18

**Authors:** Junfeng Lin, Yaxin Lv, Kai Song, Xuwei Song, Hongjun Zang, Pingwu Du, Yaping Zang, Daoben Zhu

**Affiliations:** 1 Beijing National Laboratory for Molecular Sciences, CAS Key Laboratory of Organic Solids, Institute of Chemistry, Chinese Academy of Sciences, Beijing, 100190 China; 2 University of Chinese Academy of Sciences, Beijing, 100049 China; 3 School of Chemistry, Tiangong University, Tianjin, 300387 China; 4 Hefei National Research Center for Physical Sciences at the Microscale, iChEM, CAS Key Laboratory of Materials for Energy Conversion, Department of Materials Science and Engineering, University of Science and Technology of China, Hefei, 230026 China

**Keywords:** Catalysis, Molecular electronics, Electrocatalysis

## Abstract

Electrophilic aromatic substitution (EAS) is one of the most fundamental reactions in organic chemistry. Using an oriented external electric field (OEEF) instead of traditional reagents to tune the EAS reactivity can offer an environmentally friendly method to synthesize aromatic compounds and hold the promise of broadening its scope. Despite these advantages, OEEF catalysis of EAS is difficult to realize, due to the challenge of microscopically orienting OEEF along the direction of electron reorganizations. In this work, we demonstrate OEEF-catalyzed EAS reactions in a series of cycloparaphenylenes (CPPs) using the scanning tunneling microscope break junction (STM-BJ) technique. Crucially, the unique radial π-conjugation of CPPs enables a desired alignment for the OEEF to catalyze the EAS with Au STM tip (or substrate) acting as an electrophile. Under mild conditions, the OEEF-catalyzed EAS reactions can cleave the inherently inert C(*sp*^2^)-C(*sp*^2^) bond, leading to high-yield (~97%) formation of linear oligophenylenes terminated with covalent Au-C bonds. These results not only demonstrate the feasibility of OEEF catalysis of EAS, but also offer a way of exploring new mechanistic principles of classic organic reactions aided by OEEF.

## Abstract (web summary)

Novel methodologies for cleaving inherently inert C(*sp*^2^)‒ C(*sp*^2^) bonds are desirable. Here, the authors report the use of an oriented electric field to cleave C(*sp*^2^)‒ C(*sp*^2^) in cycloparaphenylenes via electrophilic aromatic substitution.

## Introduction

Electrophilic aromatic substitution (EAS) reaction is one of the most important reactions in organic chemistry^1–4^. Since its discovery centuries ago, EAS reaction has been a general route to synthesize aromatic compounds, and fundamental studies on EAS have played a crucial role in understanding the basic principles of physical organic chemistry^5^. A well-established EAS reaction mechanism involves a rate-determining step featuring the formation of a charge-separated σ-complex intermediate along the reaction coordinate^1,6^. However, due to the relatively large activation energy for forming the charge-separated state, EAS reactions are often limited to systems containing highly activated (nucleophilic) nuclei or very strong Lewis acid catalysts^5,7^.

It has long been predicted that an oriented external electric field (OEEF) has the unique ability to control chemical reactions through stabilizing polar or charge-separated states^8–11^_._ In 2016, OEEF catalysis of Diels–Alder reactions was realized using the scanning tunneling microscopy (STM) based technique^12^, demonstrating theoretical predictions by refs. ^13,14^. Recently, many other interesting works about the OEEF catalysis of various chemical reactions have been reported, such as OEEF-catalyzed alkoxyamine homolysis^15^, cumulene isomerization^16^, and so on^17–19^. OEEF can theoretically promote the formation of the charge-separated σ-complex intermediate, thus catalyzing EAS reactions, as predicted by ref. ^2^ Using OEEF instead of traditional catalysts to manipulate the EAS process provides an environmentally friendly method to synthesize aromatic compounds. Furthermore, electrical modulation of EAS can offer a controllable platform for mechanistic studies, and, importantly, hold the promise of activating weak electrophiles, hence broadening the scope of EAS. Despite the theoretical promise, experimental demonstration of OEEF catalysis of EAS has not been reported so far due to the following challenges. First, using OEEF to catalyze a chemical reaction requires a very large electric field, which is difficult to generate in traditional experimental settings^11,20^. Second, to harness the field effect, the OEEF should be applied along the direction of electron reorganization (referred to as the reaction axis)^10,20^. Specifically, for EAS reactions, the OEEF direction has to be nearly perpendicular to the aromatic ring to promote the electrophilic attack and charge transfer, and such microscopic alignment is extremely challenging to realize in experiments.

**Fig. 1:**
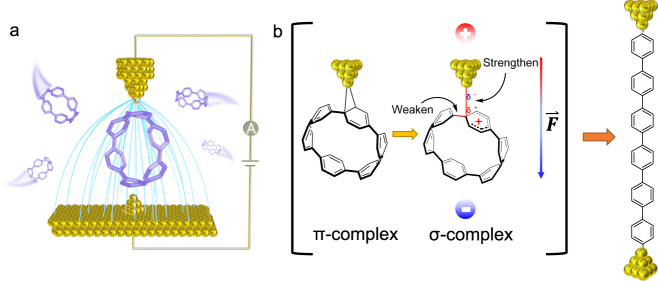
Schematic representation of OEEF catalysis of EAS reactions. **a** Schematic of single-[6]CPP junction. **b** The proposed mechanism of the OEEF-catalyzed EAS reaction which proceeds via a transition from π-complex to charge-separated σ-complex intermediate.

In this work, we use the scanning tunneling microscopy break junction (STM-BJ) technique to demonstrate OEEF-catalyzed EAS reactions in a series of hoop-shaped hydrocarbon [n]cycloparaphenylenes ([n]CPPs) (Fig. 1). STM-BJ can conveniently create a large OEEF within the nanogap between the Au STM tip and substrate by applying a tip bias voltage^11^. More crucially, by taking advantage of the unique radially π-conjugated structures of CPPs, we apply the electric field perpendicular to the aromatic ring by forming atomic Au-CPP contacts, thereby achieving the desired alignment of OEEF along the EAS reaction axis. We find that, under a very mild condition (a bias voltage of <1 V), the applied OEEF significantly accelerates the EAS by the otherwise unreactive Au electrophile (STM tip or substrate). Intriguingly, this OEEF-catalyzed EAS reaction cleaves the inherently inert C(*sp*^2^)-C(*sp*^2^) bond^21,22^ and leads to a ~97% high-yield formation of linear oligophenylenes terminated with covalent Au-C bonds. Note that the C-C bond cleavage is of great importance in synthetic chemistry but is generally very difficult to realize^23–27^. Density functional theory (DFT)-based calculations further confirm that the electric field plays a crucial role in promoting the formation of the key charge-separated σ-complex intermediate. Our work thus provides an experimental demonstration of OEEF-catalyzed EAS reactions, and shows its great potential for realizing challenging chemical transformations that are difficult to achieve using traditional methods.

## Results

### Conductance measurements

CPPs, featuring cyclically connected para phenylene units, are among the most attractive conjugated macrocycles^28–33^ and are considered the basic fragments of armchair carbon nanotubes and fullerenes^30,34^. Due to their unique radially conjugated π-orbitals, CPPs can bind to Au electrodes through direct Au-π couplings (Fig. 1a)^35^. Using the STM-BJ technique, we perform bias-dependent measurements of [n]CPPs (where n is the number of phenylene units) in a nonpolar solvent 1-chloronaphthalene with a concentration of ~0.1 mM at room temperature. In brief, we repeatedly bring an Au STM tip in and out of contact with an Au substrate in the molecular solution under an applied tip bias. After breaking the Au-Au point contact, the molecule binds to the STM tip and substrate and forms single Au-molecule-Au junctions. During this process, we continuously record the conductance distributions as a function of tip-substrate displacements.

**Fig. 2:**
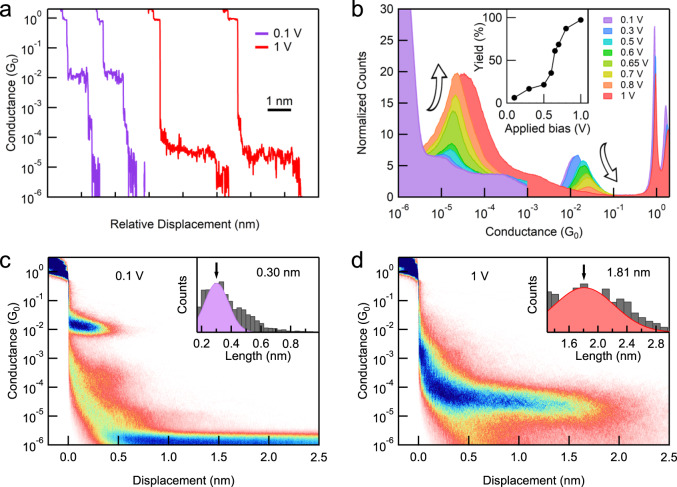
Bias-dependent STM-BJ measurement results of [6]CPP. **a** Sample conductance traces for [6]CPP measured at an applied bias of 0.1 and 1 V. **b** One-dimensional (1D) conductance histograms for [6]CPP measured at different applied biases. Inset: the yield of the Low-G junction against the applied bias. **c**, **d** Two-dimensional (2D) conductance-displacement histograms for [6]CPP measured at an applied bias of 0.1 and 1 V. Inset: relative length distribution histograms.

Figure 2a presents the sample conductance-displacement traces of [6]CPP molecules measured with different applied tip biases. At a low tip bias of 0.1 V, a short molecular conductance plateau appears at ~10^−2^ G_0_ (where G_0_ = 2*e*^2^/*h* is the conductance quantum), signifying the formation of single [6]CPP junctions driven by the direct Au-π bindings. This phenomenon is consistent with our previous observations^35^. In sharp contrast, when the applied tip bias is increased to 1 V, a much longer molecular conductance plateau shows up, and at a much lower conductance of ~5 × 10^−5^ G_0_. To better explore this phenomenon, we repeat the measurement thousands of times at each tip bias and compile these traces into logarithmically binned one-dimensional (1D) conductance histograms (Fig. 2b) and two-dimensional (2D) conductance-displacement histograms without any data selection. We also perform control measurements in pure 1-chloronaphthalene solvent to rule out the possibility of forming single solvent molecule junctions (Supplementary Fig. 1).

**Fig. 3:**
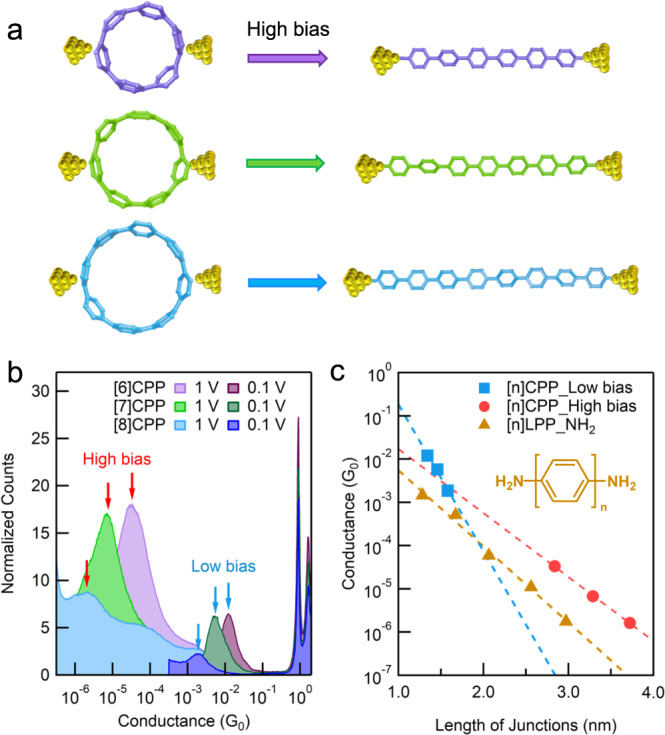
Length-dependent conductance of [n]CPPs (*n* = 6–8). **a** Schematics of EAS reactions in [n]CPPs (*n* = 6–8). **b** 1D conductance histograms for [6]CPP-[8]CPP measured at an applied low bias of 0.1 V and an applied high bias of 1 V. **c** Conductance of [6]CPP-[8]CPP determined by Gaussian fitting the conductance peaks against calculated single CPP and single LPP junction length (Au-Au distance). Conductance of the amino-anchored LPPs obtained from the previous study is included for ref. ^38^. The beta value is determined by the slope of the fitted dished lines.

At the low bias of 0.1 V, we observe a clear molecular conductance peak at 10^−2^ G_0_ in the 1D histogram. In the corresponding 2D histogram (Fig. 2c), the conductance plateau extends by ~0.3 nm, indicating a junction length of ~1.3 nm by accounting for the Au snapback length of ~1 nm in 1-chloronaphthalene solvent^36^. These characteristics confirm the repeated formation of typical Au-π bonded single [6]CPP junctions (referred to as a High-G junction)^35^. In contrast, as the applied tip bias is increased to >0.6 V, a new peak emerges at a much lower conductance of ~5 × 10^−5^ G_0_. The corresponding 2D histograms reveal a ~1.8 nm junction elongation which is ~1.5 nm longer than single-[6]CPP junctions (Fig. 2d), indicating the formation of a new type of junction (referred to as a Low-G junction). Moreover, as shown in the 1D and 2D histograms, the Low-G junction becomes more dominant as the tip bias increases. When the bias reaches 1 V, the High-G junction features almost disappear. Further clustering analysis of the traces confirms that the high bias drives the transition from the High-G junction to the Low-G junction. Note that, the yield of the Low-G junction increases by ~15-fold, from 6.25% to 97.32%, when the tip bias is increased from 0.1 to 1 V (see the inset of Fig. 2b and Supplementary Fig. 2 for the detailed data analysis). A similar transition is also observed under the large negative bias (Supplementary Fig. 3). Moreover, measurements of [7]CPP and [8]CPP show similar bias-dependent phenomena: increasing the tip bias drives the transformation from the typical single CPP junctions (High-G junctions) to a new type of Low-G junctions (Fig. 3a–b and Supplementary Figs. 4–7).

Based on the above observations, we hypothesize that the [n]CPPs undergo an EAS reaction which cleaves the C(*sp*^2^)-C(*sp*^2^) bond between phenylene units under a high tip bias, converting the hoop-shaped [n]CPP molecules to linear oligophenylenes ([n]LPPs). Moreover, the produced LPPs can directly bind to the STM tip and substrate through covalent Au-C bonds, yielding longer single-molecule junctions (which correspond to the low-G junctions observed in Fig. 2).

To rule out another possible mechanism that can yield longer single-molecule junctions, we perform control measurements of the mixed solution of [6]CPP and [7]CPP in a similar environment (Supplementary Fig. 8). Under a high tip bias, we only observe two low-G features, corresponding to the respective features shown in the pure [6]CPP and [7]CPP measurements. This result rules out the possible mechanism of a two-molecule addition reaction (such as Diels–Alder reaction and olefin metathesis reaction) that would produce a new feature corresponding to the product of the addition reaction between [6]CPP and [7]CPP.

To further validate the proposed CPP to LPP transition reaction, we first note that the measured lengths of the low-G and high-G junctions match well with the theoretical lengths of the single CPP and LPP junctions (see Supplementary Table 1 and Supplementary Fig. 9), which supports the proposed CPP to LPP transformations. We then fit the high-G and low-G conductance peaks and plot the peak conductance values against the theoretical transport length of single CPP and single LPP junctions (Fig. 3c). Note that, the conductance decreases exponentially with the length (G∝e^-*βL*^) in both the high-G and low-G series, indicating a typical coherent tunneling transport mechanism^37^. By further fitting these data, we obtain the decay constant *β* for the two types of junctions. The *β* value of the high-G series is consistent with that observed in previous work^35^ and confirms the formation of single CPP junctions, while the low-G series shows a smaller *β* value. Notably, the low-G *β* value agrees well with benchmark amine-terminated LPPs^38^ (see the brown line in Fig. 3c), confirming that the low-G junctions indeed form across LPP backbones. Furthermore, the conductance of the low-G series is higher than that of the amine-terminated LPPs, which is attributed to its lower contact resistance (which is the Y-intercept of the fitted curve) arising from the highly conducting covalent Au-C contacts^39–48^. The formation of the covalent Au-C contacts is further confirmed by comparing the conductance of the low-G series with that of the covalent Au-C bonded short benzene molecule (see Supplementary Fig. 10), and rationalized through DFT-based transport calculations (see Supplementary Fig. 11 for detailed discussions). It is worth noting that covalent Au-C bonding contacts have long been pursued because they yield stable and highly conductive metal-molecule interfaces^39–48^. Previous methods for constructing such contacts typically rely on terminal anchor groups such as alkynes^42,46–48^, iodine^43^, diazonium^44,45^, trimethyltin^39,40^, or trimethylsilyl^41^ groups. This work offers a new method complementary to the reported ones for building covalent Au-C contacts.

### Mechanistic analysis

**Fig. 4:**
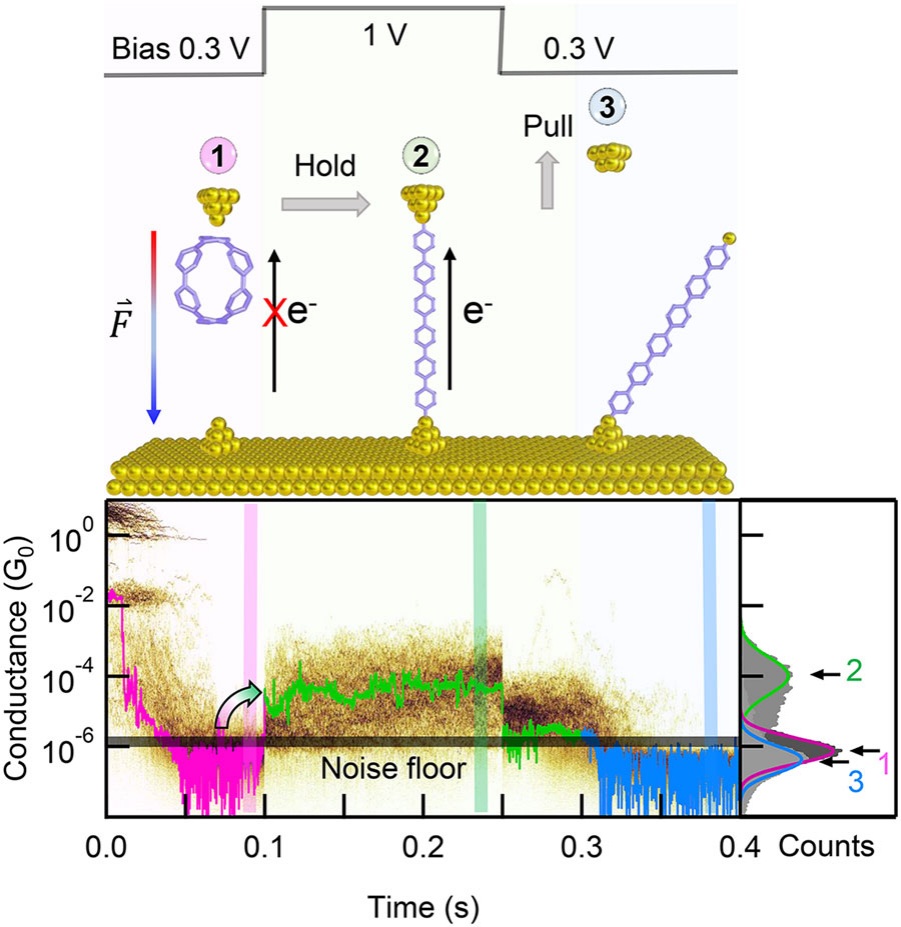
Modified STM-BJ measurements for [6]CPP. The 2D conductance-time histogram is compiled from selected traces that show a switch from noise floor to characteristic conductance of molecule under the high bias during the hold period. The conductance profiles are determined from the three regions in the 2D histograms.

We now explore the mechanism behind the observed reactions. We first perform control experiments and rule out two common mechanisms that can drive a chemical reaction under a high bias, namely, electrochemical redox-driven process^38,43–45,49^ and tunneling electron-driven process^50–52^. Specifically, we perform in situ cyclic voltammetry (CV) measurements of CPPs in the STM-BJ setup (see Supplementary Fig. 12). Since no obvious redox signal occurs over a large bias range between −0.8 and 1.2 V, an electrochemical redox mechanism is excluded. To identify the effect of tunneling electrons, we perform modified STM-BJ measurements of [6]CPP (see Fig. 4 and Supplementary Fig. 13). Specifically, under the low tip bias (0.3 V), we withdraw the STM tip until the single [6]CPP junction breaks and the current drops to the instrumental noise floor of the STM-BJ. We then hold the STM tip to maintain a tip-substrate nanogap for 250 ms, during which a high bias of 1 V is applied for 150 ms. Interestingly, we observe that the applied high bias can drive the conductance jumping to ~5 × 10^−5^ G_0_, indicating the in situ formation of single LPP junctions. This is evidenced by the conductance profiles of the 2D conductance-time histograms. As this CPP to LPP reaction happens without initial current, a tunneling current-driven reaction mechanism is eliminated.

Notably, the cleavage of the nonpolar C(*sp*^2^)-C(*sp*^2^) bond is a major challenge due to its inherent inertness. We deduce that, during the STM-BJ measurements, the large OEEF generated between the tip-substrate nanogap plays a critical role in promoting the observed cleavage of the C-C bond in CPPs. Further analysis of the experimental data indicates that the reaction rate constant is increased by ~50-fold with the increase of the tip bias from 0.1 to 1 V (see Supplementary Fig. 14 for detailed information). As the solvent polarity plays a crucial role in determining the electric field distribution, we perform additional control measurements of [6]CPP in another nonpolar solvent 1,2,4-trichlorobenzene (TCB) and a polar solvent propylene carbonate (PC) which can screen the electric field. In TCB, we observe a similar CPP to LPP transition (Supplementary Fig. 15). By contrast, there is no stable molecular junctions formed in PC solvent (Supplementary Fig. 16), which indicates that the polar solvent is unfavorable for the CPP-Au binding and the reactions. These control measurements further confirm the crucial role of the electric field played in catalyzing the reactions.

**Fig. 5:**
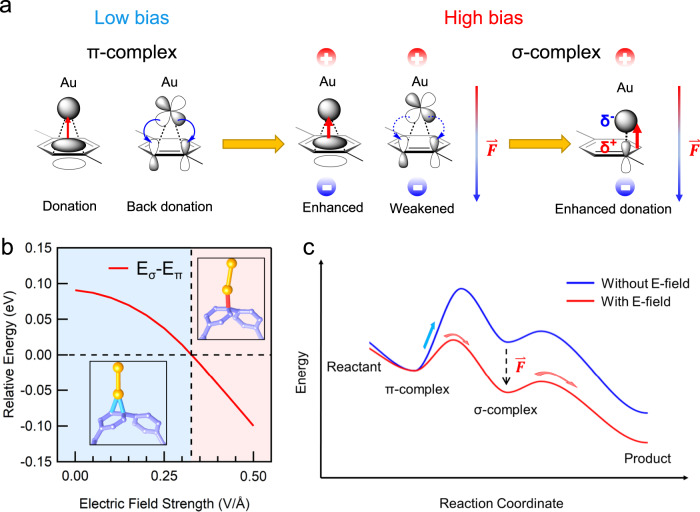
Proposed mechanism of OEEF-catalyzed EAS. **a** Electric field effect on orbital interactions between CPP molecule and Au. **b** Relative energy of π-complex and σ-complex against the strength of OEEF, where E_σ_ is the energy of σ-complex, and E_π_ is the energy of π-complex. (See Supplementary Fig. 17 for the optimized geometries under different electric fields). **c** Schematic of the energy profile of OEEF-catalyzed EAS reaction. The existence of OEEF facilitates the transition from the π-complex to the σ-complex.

Specifically, CPPs undergo EAS reactions catalyzed by the electric field, with Au acting as an electrophile. We first point out that the initial Au-π binding between CPPs and Au electrodes leads to the formation of a stable π-complex intermediate. More importantly, in this π-complex intermediate, the EAS reaction axis perfectly aligns along the direction of the OEEF, which is critical for harnessing the electric field effect on the EAS^10,20^. It should be further noted that, the Au-CPP interaction in the π-complex involves two major components: electron donation from the filled π-orbital of CPP to the 6*s* orbital of the Au atom, and back-donation from the filled 5*d* orbital of the Au atom to the empty CPP π*-orbital^35^ (Fig. 5a, left). In this scenario, when an OEEF is applied along the Au-π binding axis, the electron donation is enhanced while the back-donation is weakened (Fig. 5a, middle), thereby increasing the charge separation within the complex. This effect can eventually lead to the transition from the π-complex to the charge-separated σ-complex (Fig. 5a, right), which is known as the key intermediate of the EAS reaction.

### DFT calculations

This transition process can be further rationalized through DFT calculations using the Perdew–Burke–Ernzerhof (PBE) exchange-correlation functional implemented by the Fritz Haber Institute ab initio molecular simulation (FHI-aims) packages^53–55^. The all-electron numeric atom-centered basis set is used (see SI for the calculation details). As shown in Fig. 5b, in the absence of an OEEF (or under a small OEEF), the π-complex is much more stable than the σ-complex, supporting the observed formation of Au-π bonded single CPP junctions at the low tip bias. In contrast, as the OEEF is increased to >~3 V/nm, the σ-complex becomes the more stable intermediate, evidencing the proposed OEEF-driven transition from the π-complex to the σ-complex. Once the charge-separated σ-complex intermediate is formed, the nearby C-C bond is cleaved spontaneously, yielding a positively charged phenylene radical that rapidly binds to another Au electrode (cathode) rather than the nearby one due to the electric field and strain effects. These reactions thus lead to the formation of long LPP junctions with two terminal covalent Au-C bonds. This suggests that OEEF can be a general catalyst for accelerating the EAS reactions via promoting the formation of the σ-complex key intermediate (see the schematic diagrams shown in Fig. 5c). Normally, the electrophilic substitution of a benzene ring leads to the cleavage of C-H bonds. In contrast, our results show unusual selectivity towards breaking the more inert C-C bond. This unique reaction site selectivity is attributed to the strained structures of the CPPs, where the release of the strain energy provides the driving force for the highly selective EAS reactions. This result offers an unprecedented route to activate C-C bonds to achieve versatile carbon skeletons.

In conclusion, we have experimentally demonstrated OEEF catalysis of fundamental EAS reactions using the STM-BJ technique. These reactions selectively cleave the highly inert nonpolar C(*sp*^2^)-C(*sp*^2^) bond of a series of CPPs under very mild reaction conditions (a bias voltage of <1 V), allowing the high-yield (~97%) formation of LPPs with terminal covalent Au-C bonds. This study not only expands the scope of EAS via activating otherwise unobtainable electrophilic attack aided by OEEF, but also pushes the frontier of mechanistic understanding of fundamental chemical reactions and electrocatalysis.

## Methods

### STM-BJ measurements

All [n]CPP (*n* = 6–8) molecules were purchased from Tokyo Chemical Industry (TCI), and directly used without further purification. Single-molecule conductance measurements were carried out using a custom-built scanning tunneling microscope-break junction (STM-BJ) platform. All the STM-BJ experiments were performed in dilute [n]CPP solutions (~0.1 mM concentration) under ambient conditions and at room temperature. During the conductance measurements, the Au tip (99.999% purity) and the substrate coated with a gold layer were used as the two electrodes. Under an applied tip bias voltage of 0.1 to 1 V, the Au tip was driven to move in and out of contact with the substrate deposited with [n]CPP solutions to form and break Au-CPP-Au molecule junctions. During this process, the current was recorded continuously and the conductance was calculated by the formula G = I/V. One-dimensional conductance histograms and two-dimensional conductance histograms were constructed by compiling >3000 collected conductance traces without any data selection.

### DFT-based calculations

The geometry optimization and molecular total energy calculation were performed using the Perdew–Burke–Ernzerhof (PBE) exchange-correlation functional implemented by the Fritz Haber Institute ab initio molecular simulation (FHI-aims) packages. The calculations were performed without and with an external homogeneous electric field. The Landauer transmission across these junctions was calculated using the nonequilibrium Green’s function (NEGF) formalism.

## Supplementary information

Supplementary Information

## Data Availability

The main data supporting the findings of this study can be found in the manuscript and the Supplementary Information file.
