# Supplementary material for: Cleavage of non-polar C(sp2)‒C(sp2) bonds in cycloparaphenylenes via electric field-catalyzed electrophilic aromatic substitution
Source: Nat Commun. 2023 Jan 18;14:293. doi: 10.1038/s41467-022-35686-4 (PMC9849230; doi:10.1038/s41467-022-35686-4)
Supplement: Supplementary file 1 — Supplementary Information [file 41467_2022_35686_MOESM1_ESM.pdf]

Supplementary Information

Cleavage of non-polar C(sp2)–C(sp2) bonds in cycloparaphenylenes via electric field-catalyzed electrophilic aromatic substitution

Junfeng Lin, Yaxin Lv, Kai Song, Xuwei Song, Hongjun Zang, Pingwu Du, Yaping Zang*, Daoben Zhu

Email: zangyaping@iccas.ac.cn

Table of Contents:

1. Supplementary Methods

2. Supplementary Discussions

3. Supplementary Notes

4. Supplementary References

1. Supplementary Methods

1.1. Molecular junction optimization and transmission calculations

We attach two Au clusters containing 4 Au atoms (Au-Au bond length is constrained to 2.88 Å) to the two sides of the optimized molecular structure and relax the junction geometries using the Perdew-Burke-Ernzerhof (PBE) exchange-correlation functional implemented by the Fritz Haber Institute ab initio molecular simulation (FHI-aims) packages. After relaxation, the 4 atom Au clusters are replaced by Au pyramids containing 60 Au atoms in 6 layers. The Landauer transmission across these junctions is calculated using the nonequilibrium Green's function (NEGF) formalism.

1.2. Molecular junction optimization under electric field

We first attach one Au cluster containing 2 Au atoms to the optimized [6]CPP molecule to form an Au-CPP π-complex and relax its geometry using the PBE exchange-correlation functional implemented by the FHI-aims packages, all-electron numeric atom-centered basis set (light computational settings) is used. After geometry optimization, we apply an external homogeneous electric field with orientation aligned along the direction of Au-π bond. We relax the Au-CPP complex geometries under electric field of different strength between 0.05 to 0.5 V/Å.

2. Supplementary Discussions

2.1. Control STM-BJ measurements in pure 1-chloronaphthalene solvent

We have performed control STM-BJ measurements in pure 1-chloronaphthalene solvent, and did not observe any obvious molecular conductance features (see Figure R3). This confirms that the observed single molecule junction signatures arise from the CPP molecules instead of the solvent.

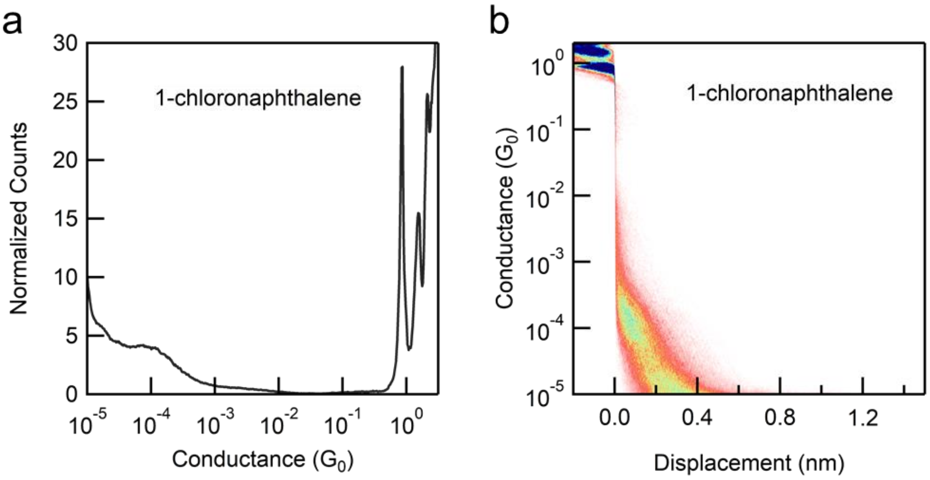

Supplementary Fig. 1. 1D and 2D histograms for 1-chloronaphthalene solvent. (a) 1D histograms for pure 1-chloronaphthalene solvent measured at an applied bias of 0.1 V. (b) 2D histogram for pure 1-chloronaphthalene solvent measured at an applied bias of 0.1 V.

2.2. Data clustering for [6]CPP

To gain more quantitative information of the high bias induced transition from the High-G junction to the Low-G junction, we sort the conductance traces measured at each bias into three categories, corresponding to the High-G junction (II), the Low-G junction (III) and the direct tunneling nanogap (IV), see Figure S2. We note that the probability of junction formation is around ~50% for all the measurements. Moreover, the Low-G junction yield (Y_{Low-G}), determined by the ratio of the number of the Low-G junction traces to the total numbers of junction traces, increases with the increase of the applied tip bias. More specifically, at the low bias of 0.1 V, the Y_{Low-G} is as low as 6.25%. In sharp contrast, the Y_{Low-G} increases by ~15-fold and reaches 97.32% at the high bias of 1 V. These results indicate that the increase of the high bias to 1 V leads to an almost complete transition from the High-G junction to the new type of Low-G junction.

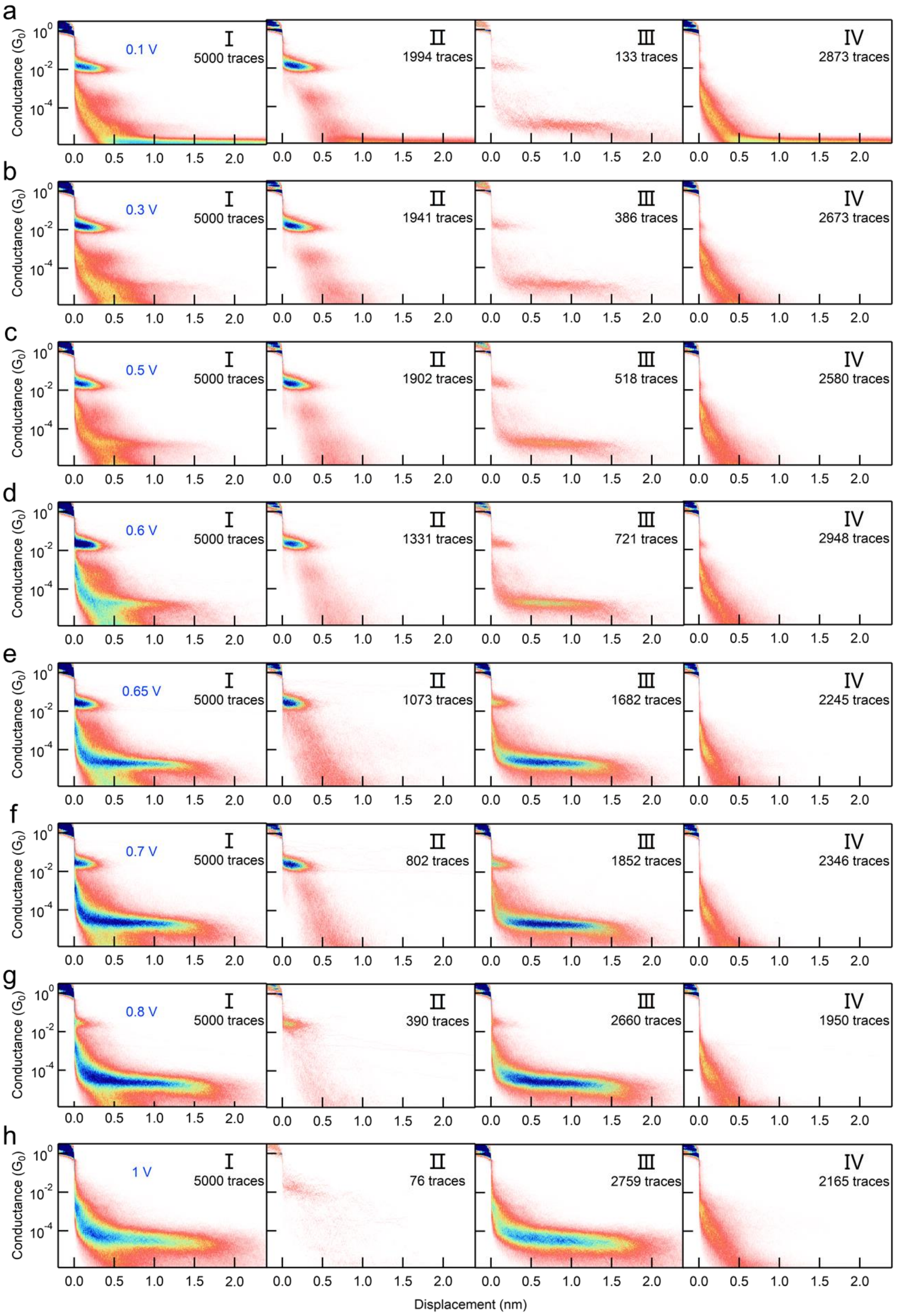

Supplementary Fig. 2. 2D conductance histograms for [6]CPP. (a-h) 2D histograms compiled from traces for [6]CPP measured at different applied bias. Panel I: 2D histograms compiled from the total 5000 traces. Panel II: 2D histograms compiled from the sorted traces with the High-G feature corresponding to the single [6]CPP junction. Panel III: 2D histograms compiled from the sorted traces with the Low-G feature. Panel IV: 2D histograms compiled from the direct tunneling traces with exponentially decayed conductance.

2.3. STM-BJ experiment under negative bias.

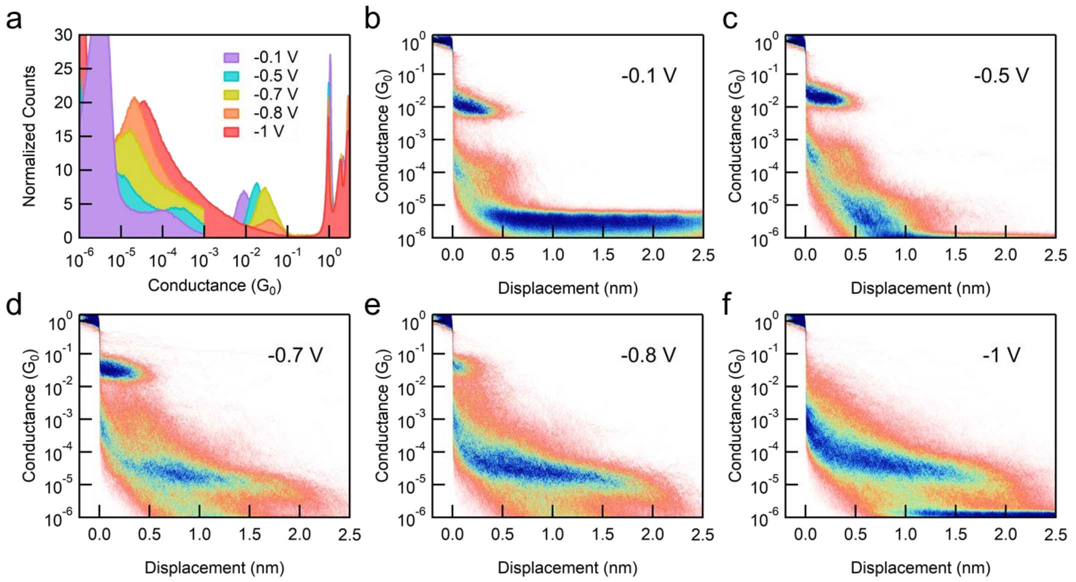

Supplementary Fig. 3. 1D and 2D histograms for [6]CPP measured at negative biases. (a) 1D conductance histogram for [6]CPP under different negative biases. (b-f) 2D conductance histograms for [6]CPP under different negative biases. Under negative bias, the catalysis effect of electric field is the same as the positive bias condition because of the symmetry of the CPP junction.

2.4. Additional 1D and 2D conductance histograms for [n]CPPs

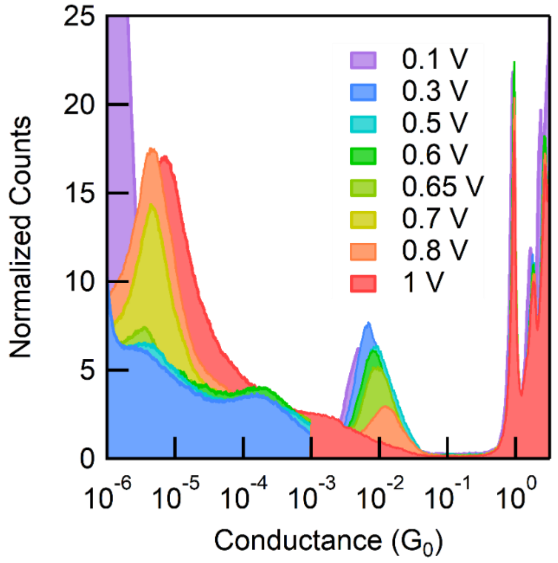

Supplementary Fig. 4. 1D conductance histograms for [7]CPP measured at different applied biases. Each histogram is compiled from 3000 traces without any data selection.

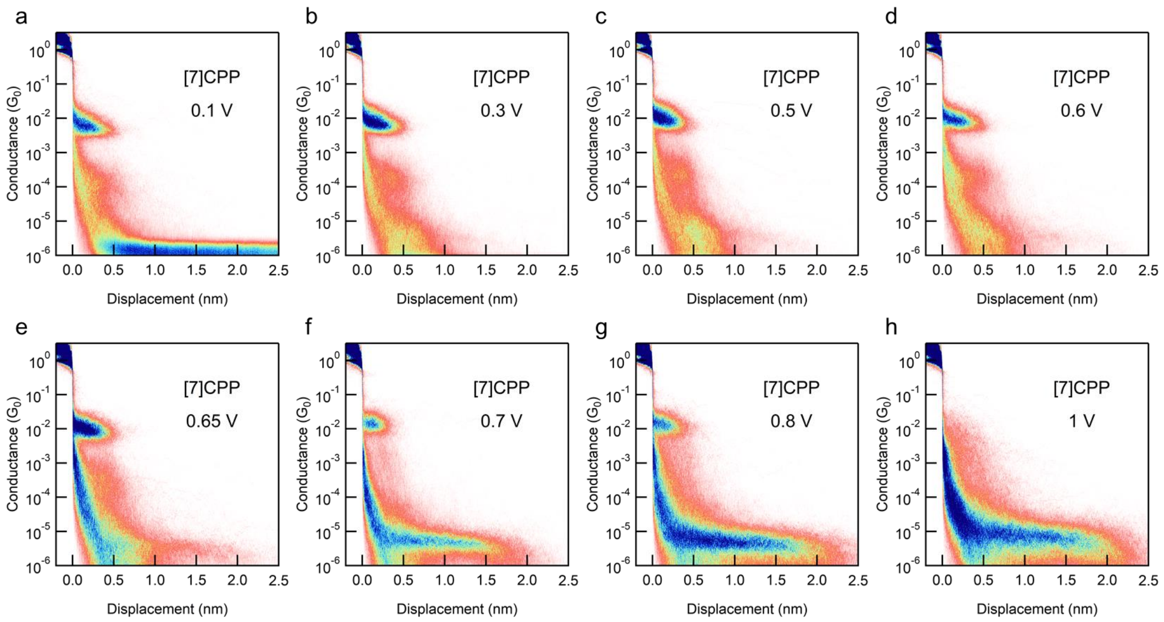

Supplementary Fig. 5. 2D conductance histograms for [7]CPP measured at different applied biases. (a-h) 2D histograms measured at a bias of 0.1 V-1 V. Each histogram is compiled from 3000 traces without any data selection.

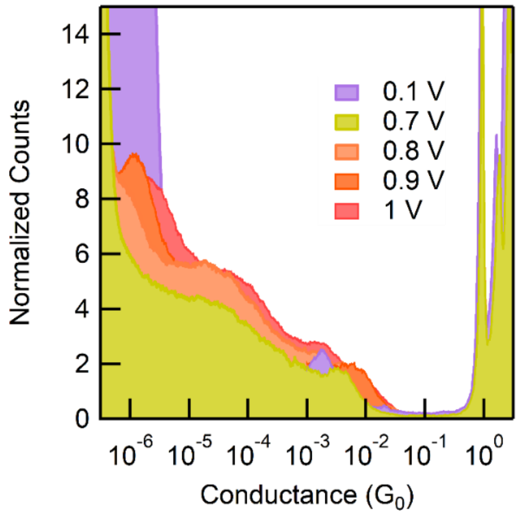

Supplementary Fig. 6. 1D conductance histograms for [8]CPP measured at different applied biases. The junction formation probability of [8]CPP is lower than [6]CPP and [7]CPP because of its lower strain in the macrocycle. Each histogram is compiled from ⩾3000 traces without any data selection.

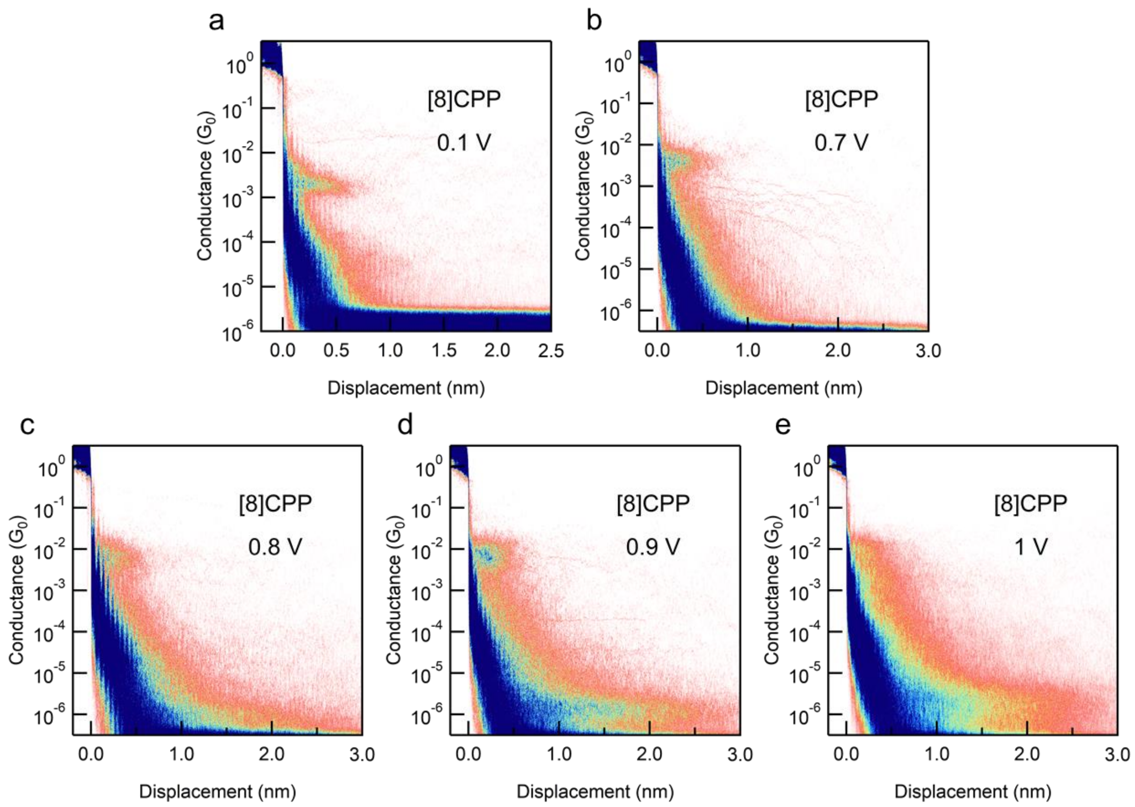

Supplementary Fig. 7. 2D conductance histograms for [8]CPP measured at different applied biases. (a-e) 2D histograms measured at a bias of 0.1 V-1 V. Each histogram is compiled from ⩾3000 traces without any data selection.

2.5. Mixed [6]CPP and [7]CPP solution measurements

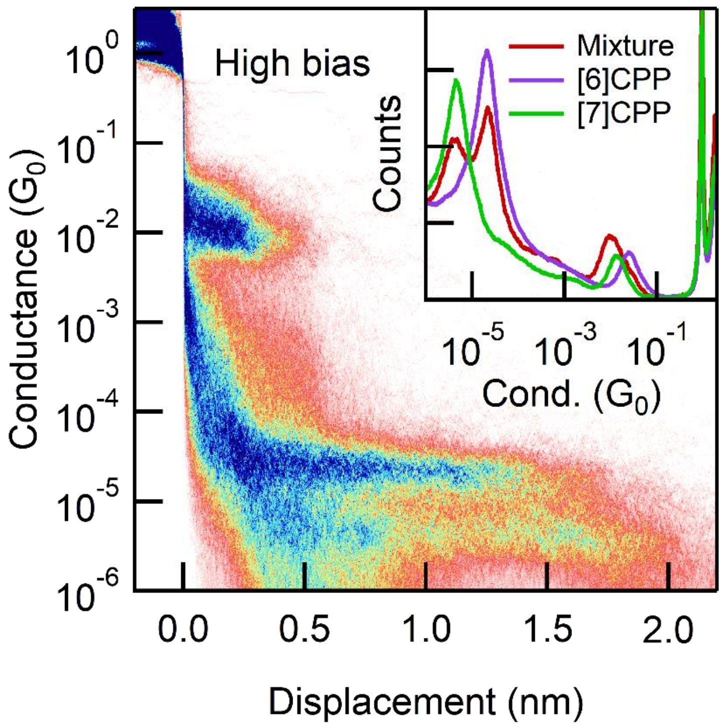

Supplementary Fig. 8. 2D conductance histogram for measurements of the mixed [6]CPP and [7]CPP solution. Inset: Corresponding 1D conductance histograms for measurements of the mixed solution of [6]CPP and [7]CPP (red line), and pure solution of [6]CPP (purple line) and [7]CPP (green line). Under a high tip bias, there are only two Low-G features, which correspond to those shown in the pure [6]CPP and [7]CPP measurements. This result rules out one possible mechanism of a two-molecule addition reaction (such as Diels–Alder reaction and olefin metathesis reaction) that would produce a new feature corresponding to the product of the addition reaction between [6]CPP and [7]CPP.

2.6 Junction length analysis

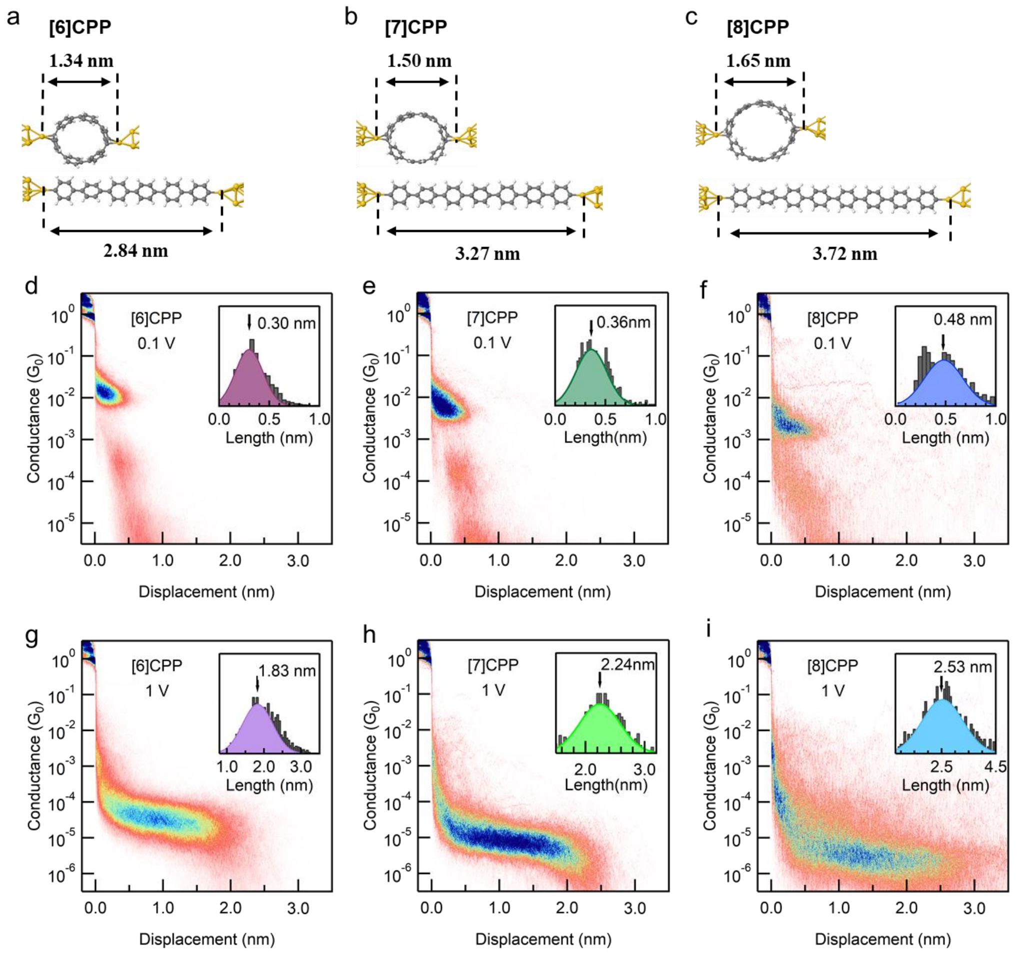

Supplementary Fig. 9. Junction length analysis of [n]CPPs (n=6-8). (a-c) DFT-optimized geometries of single CPP and LPP junctions. (d-f) 2D conductance histograms for [6]CPP-[8]CPP measured at an applied tip bias of 0.1 V. (g-i) 2D conductance histograms for [6]CPP-[8]CPP measured at an applied tip bias of 1 V. The direct tunneling traces with exponentially decayed conductance are excluded for constructing the 2D histograms. Inset: Relative length distribution histograms. The calculated junction length and the measured molecular conductance plateau extension length are summarized in Table S1 below.

2.7. Fitting of length-dependent conductance for Low-G

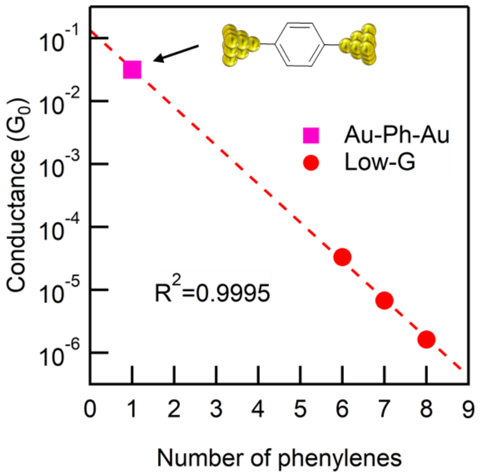

Supplementary Fig. 10. Conductance versus number of phenylene units. The conductance data for the covalent Au-C bonded benzene junction (pink square) is taken from reference[1]. R^2 refers to the coefficient of determination, and an R^2 close to 1 indicates nearly perfect data fitting. This suggests that our experiments indeed produce linear oligophenylenes covalently connected to Au electrodes through Au-C bonds.

Supplementary Table 1. Experimental and calculated junction length. We can see that the measured length difference between the High-G junction (0.1 V) and Low-G junction (1 V) matches very well with the theoretical length difference between single CPP junction and single LPP junction. Notice that, due to the Au electrode snapback distance, the absolute value of the measured length is about ~1 nm shorter than the theoretical results. These support the hypothesis of high bias driven transition from the High-G junction to the Low-G junction.

**Molecule**	**Experiment**			**DFT calculation**		
	Length of [n]CPP in 0.1 V	Length of [n]CPP in 1 V	Difference	Length of [n]CPP junction	Length of [n]LPP junction	Difference
[6]CPP	0.30 nm	1.83 nm	1.53 nm	1.34 nm	2.84 nm	1.50 nm
[7]CPP	0.36 nm	2.24 nm	1.88 nm	1.50 nm	3.27 nm	1.77 nm
[8]CPP	0.48 nm	2.53 nm	2.05 nm	1.65 nm	3.72 nm	2.07 nm

2.8. Calculated transmissions of single [n]CPP and [n]LPP junctions

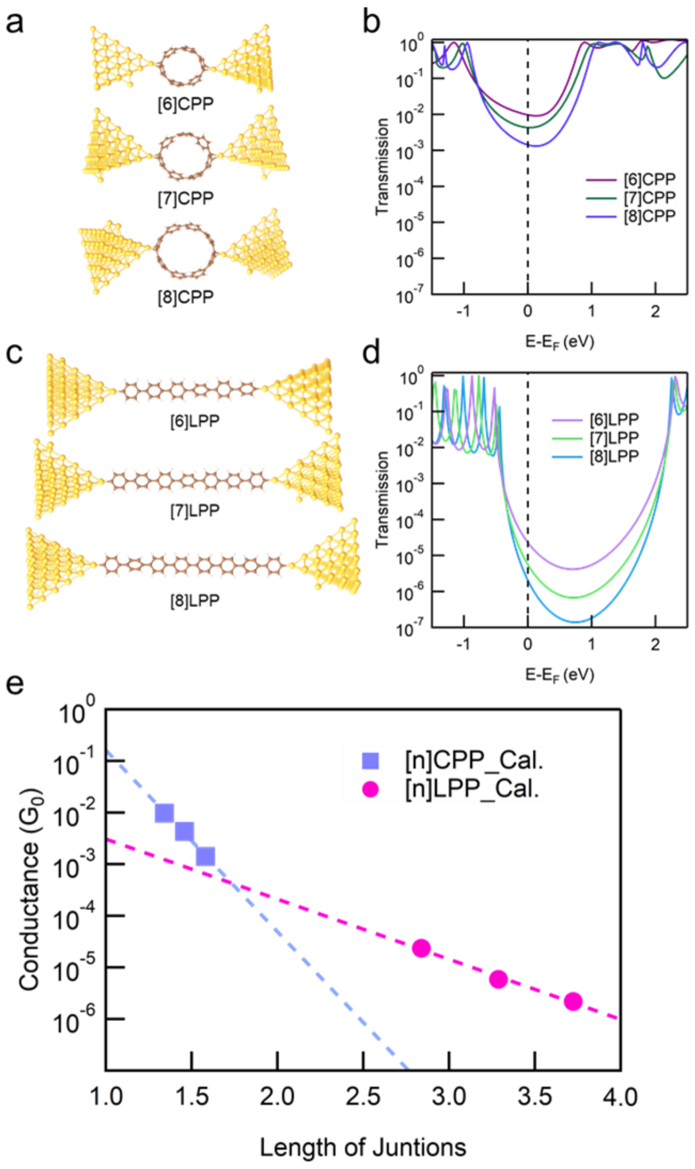

Supplementary Fig. 11. Transmission calculations of [n]CPPs and [n]LPPs (n= 6,7,8). (a) Junction structures of [6]CPP-[8]CPP used for transmission calculations. (b) Transmission functions against energy for single [6]CPP-[8]CPP junctions. (c) Junction structures of [6]LPP-[8]LPP used for transmission calculations. (d) Transmission functions against energy for single [6]LPP-[8]LPP junctions. (e) Calculated conductance against transport length (Au-Au distance) for single CPP and LPP junctions. The CPP series has a higher conductance and a larger decay constant β than the LPP series, in good agreement with experimental results shown in Figure 3c. These results thus support the proposed transitions from CPP to LPPs.

2.9. In situ cyclic voltammetry of [n]CPP

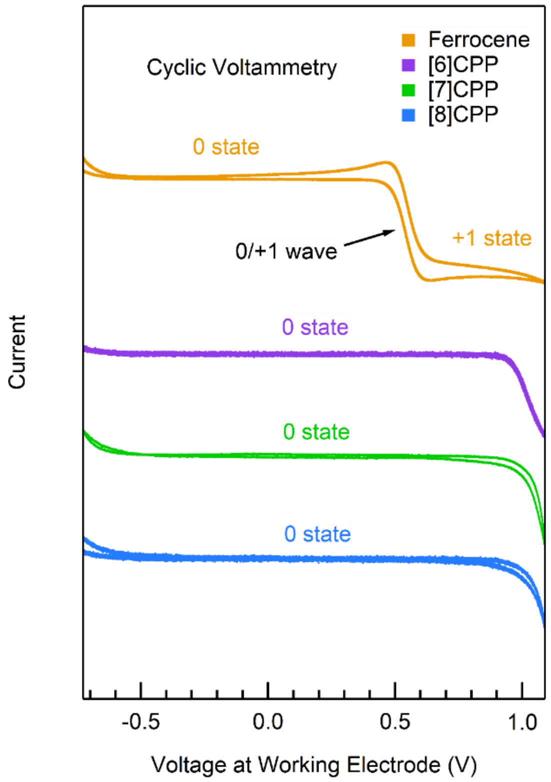

Supplementary Fig. 12. In situ cyclic voltammetry (CV) plots. CV measurements are performed in a ~0.5 mM solution of [n]CPPs (n= 6,7,8) and ferrocenes in propylene carbonate (PC) solvent containing 0.1 M of supporting electrolyte, tetrabutylammonium perchlorate (TBAP). Current flowing into the wax-coated STM tip is measured while a linear voltage sweep (100 mV/s) is applied to the substrate. In the applied bias range between -0.8 V and -1.2 V, ferrocene shows a clear oxidative wave at about 0.5 V while [n]CPP (n=6,7,8) do not show any obvious redox signal wave. These results demonstrate that the break of C-C bond in [n]CPP does not relate to redox event, thus rules out an electrochemical mechanism.

2.10. Modified STM-BJ measurements for [6]CPP

As discussed in the manuscript and shown in Figure 4, the applied high bias can drive the conductance jump from instrumental noise floor to the signature conductance of single LPP junctions. Here, we perform similar modified STM-BJ measurements where we first form the single CPP junction at a low bias and ramp the bias up. As shown in Figure S14, the applied high bias can drive the formation of single LPP junctions.

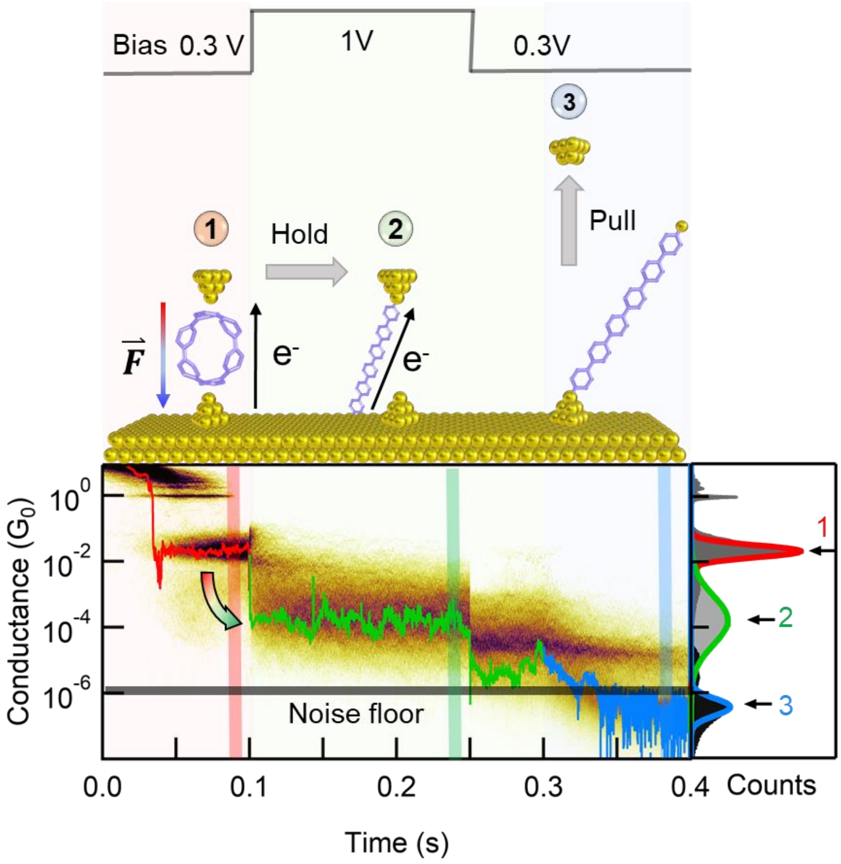

Supplementary Fig. 13. Modified STM-BJ measurements for [6]CPP. The 2D conductance-time histograms is compiled from selected traces that show a switch from the High-G to the Low-G under the high bias during the hold period. The conductance profiles are determined from the three regions in the 2D histograms. These results indicate a high bias induced in situ transition from single CPP junctions to single LPP junctions.

2.11. First-order kinetic analysis for [6]CPP

We use the simple first-order reaction model to do some rough kinetic analysis [2]:

$$\frac{d[c]}{dt} = -k[c] \tag{1}$$

where $[c]$ is the concentration of the reactant, and k is the rate constant (in units of 1/time). Notice that our reactions only happen at the nanogap between the STM tip and substrate, and so $[c]$ here should be interpreted as a local concentration measure. To determine k in the above equation, we only need to measure $[c]_t$ at two time instances. Here we choose t=0 and t=0.015s, and determine k via:

$$k = -ln\left(\frac{[c]_t}{[c]_0}\right)/t \tag{2}$$

We approximate $[c]_t$ by the ratio of the number of the Low-G junction to the total number of junction traces. We can then determine k under different biases (see Figure R8). We see that k is increased from 4.3 s^{-1} at 0.1 V to 241.3 s^{-1} at 1 V, implying a ~50-fold increase of reaction rate.

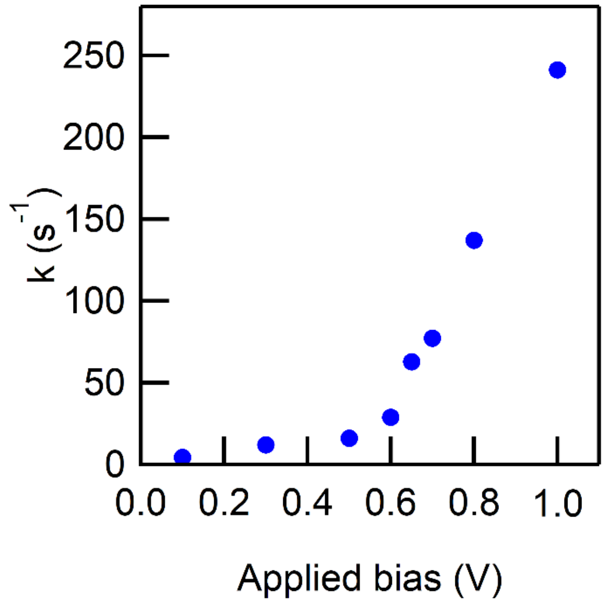

Supplementary Fig. 14. Rate constant against applied bias for [6]CPP. The rate constant increases from 4.3 s^{-1} under 0.1 V to 241.3 s^{-1} under 1 V.

2.12. STM-BJ measurements for [6]CPP in TCB solvent

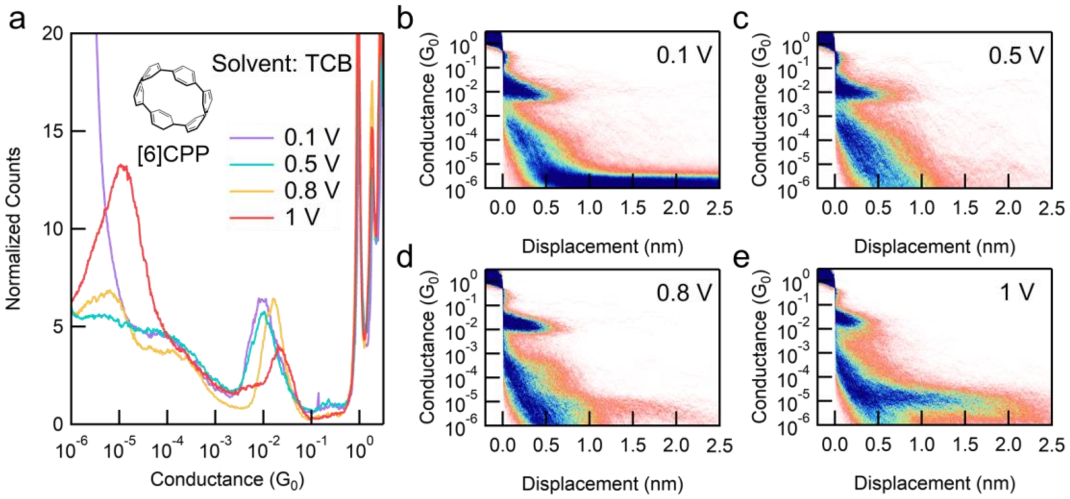

Supplementary Fig. 15. STM-BJ measurements for [6]CPP in TCB solvent. (a) 1D conductance histogram for [6]CPP in TCB solvent under different biases. (b-e) 2D conductance histograms for [6]CPP in TCB solvent under different biases. In TCB solvent, a similar CPP to LPP transition is observed but the bias needed for catalyzing the EAS reaction is higher (~1 V vs ~0.6 V in 1-chloronaphthalene).

2.13. STM-BJ measurements for [6]CPP in PC solvent

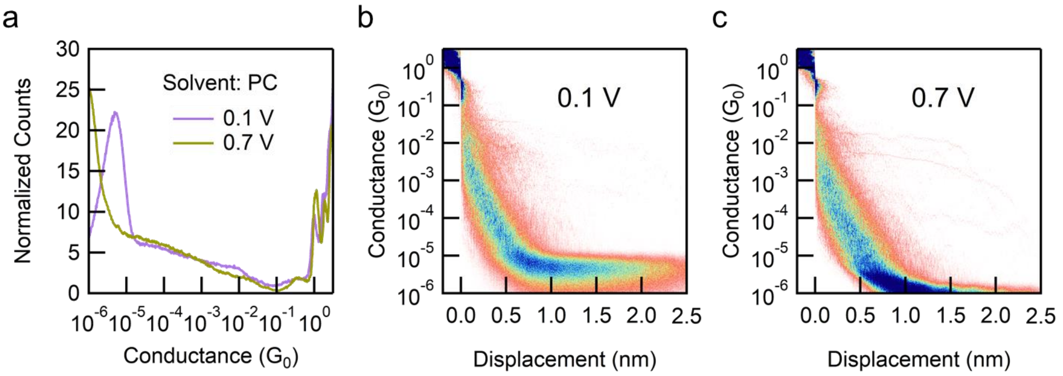

Supplementary Fig. 16. STM-BJ measurements for [6]CPP in PC solvent. (a) 1D conductance histogram for [6]CPP in PC solvent under different biases. (b-c) 2D conductance histograms for [6]CPP in PC solvent under different biases. Both 1D and 2D histograms show there is no stable molecular junctions formed in PC solvent.

2.14. Total energy calculations of Au-[6]CPP complex under electric field

Using the calculation method mentioned before, we obtain a series of optimized geometries for [6]CPP under different electric field (Figure S17). We can see that, as the applied electric field increases, the π-complex gradually converts to a more charge-separated σ-complex. To better understand the electric field driven conversion, we calculate the single point energy of the π-complex (Figure S17b) and the σ-complex (Figure S17l) under the electric field strength between 0 V/Å to 0.5 V/Å. We then obtain the field strength dependent energy difference between the π-complex and the σ-complex (Figure 5b). We can see that, as the electric field is increased to > ~0.3 V/Å, the σ-complex becomes more stable than the π-complex as its total energy becomes lower. These results thus rationalize the proposed mechanism of OEEF-driven transition from the π-complex to the σ-complex underlying the EAS process.

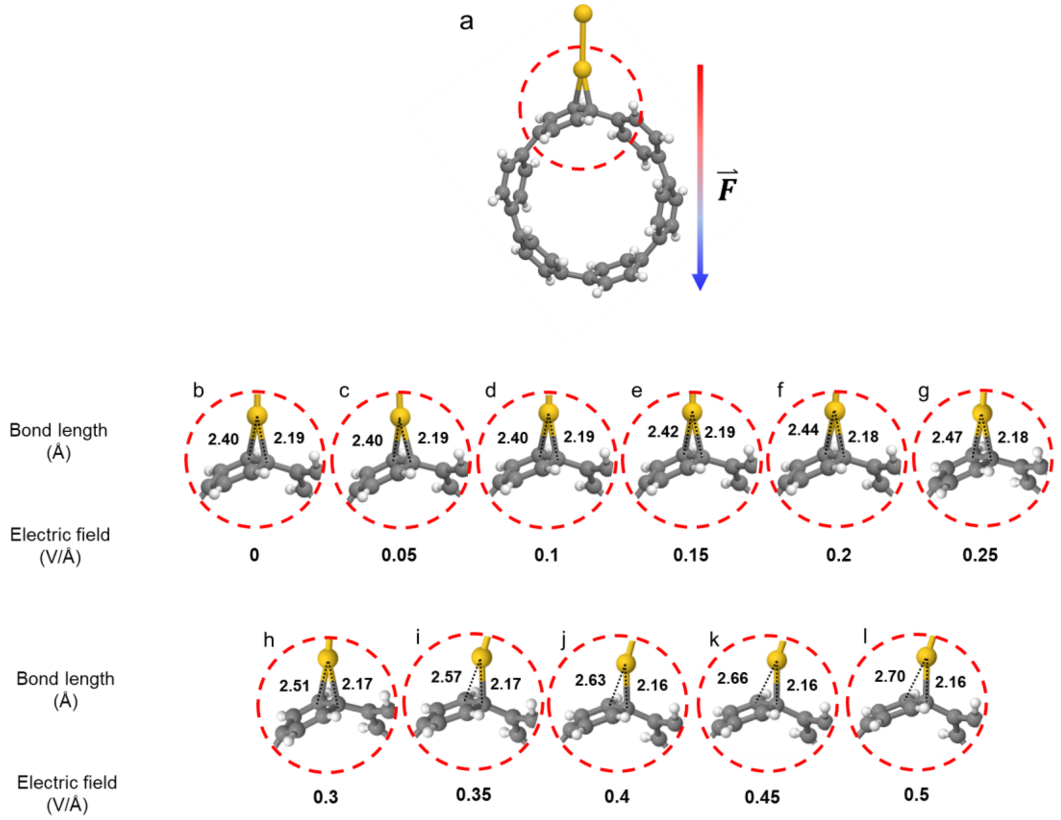

Supplementary Fig. 17. Optimized geometries of Au-CPP complex under different electric fields. (a) Input geometry of Au-CPP π-complex, and its orientation along the applied electric field. (b)-(l) The optimized geometries obtained under different electric

field intensities. We can see that as the electric field increases, the π-complex gradually transforms into the charge-separated σ-complex.

3. Supplementary Notes

Cartesian Coordinates of Optimized Structures.

Supplementary Table 2. [6]CPP π-complex (F= 0 V/Å)

PBE/ all-electron numeric atom-centered basis, light computational settings

Total energy of the DFT calculation: -1109013.311626822 eV

Atom	x	y	z
C	-0.66824086	-3.50452639	1.77774216
C	-1.87179141	-2.77342898	1.73361373
C	-2.63060989	-2.71340593	0.56896068
C	-2.22400759	-3.39748018	-0.59043045
C	-1.18384394	-4.34007178	-0.45650148
C	-0.41870726	-4.39162062	0.70701920
C	-3.77648475	-1.97421402	-2.06210948
C	-3.70127449	-0.73399956	-2.72650768
C	-2.49123160	-0.28624595	-3.27780155
C	-1.45106027	-1.24534189	-3.42461077
C	-1.49831872	-2.45657687	-2.76897622
C	-2.60466145	-2.80208566	-1.90898136
C	2.44850219	-1.69496113	2.55753664
C	2.00385802	-0.84108746	3.58807903
C	0.90274492	-1.29138197	4.35084572
C	0.10937225	-2.34463384	3.90215587
C	0.39232367	-2.99276443	2.67955368
C	1.65943106	-2.75042352	2.11194097
C	2.38750298	0.58879964	3.49996789
C	3.57704108	1.02918064	2.87683572
C	3.60107294	2.21252943	2.14249170
C	2.43685929	3.00141702	2.00371400

C	1.37867637	2.72193416	2.89314497
C	1.35438739	1.54053060	3.62581188
C	1.02244691	4.63465752	0.66040958
C	2.09809295	3.72230001	0.75318043
C	2.55481062	3.16310015	-0.45828678
C	1.79029560	3.24633403	-1.61667320
C	0.53724103	3.89318524	-1.61264045
C	0.25494838	4.71531681	-0.49879408
C	-1.00987286	1.59225768	-4.12507271
C	-0.23769220	2.67153214	-3.70426769
C	-0.51955617	3.33118032	-2.48663645
C	-1.77617993	3.06833295	-1.90163270
C	-2.55131801	1.99509564	-2.32569862
C	-2.10413282	1.13989721	-3.35400427
H	-2.11911271	-2.09751706	2.55282202
H	-3.45171263	-1.99799747	0.51711876
H	-0.91622193	-4.97702555	-1.30183505
H	0.43974547	-5.06473937	0.74711436
H	-4.65427432	-2.17479201	-1.44415591
H	-4.57225877	-0.07785623	-2.70110527
H	-0.52583727	-0.96049990	-3.92561245
H	-0.61051056	-3.08895854	-2.75372509
H	3.32055013	-1.41458559	1.96571542
H	0.59703905	-0.74272672	5.24368937
H	-0.79964507	-2.59573551	4.45230129
H	1.94273082	-3.25963405	1.18980731
H	4.45941219	0.38635670	2.87025080
H	4.50170701	2.46587202	1.57989569
H	0.47299290	3.32842636	2.86005905

H	0.43106013	1.26818187	4.13801081
H	0.71242886	5.20324194	1.53912530
H	3.40970652	2.48622865	-0.44987862
H	2.07757294	2.63281118	-2.47132520
H	-0.63634569	5.34578399	-0.49936572
H	-0.70352155	1.03817375	-5.01428192
H	0.65702606	2.93405206	-4.27163949
H	-2.05957464	3.58275419	-0.98296549
H	-3.41585408	1.70909678	-1.72540551
Au	-3.77116920	-4.02112761	-3.30950086
Au	-4.85961960	-6.16328250	-4.90397039

Supplementary Table 3. [6]CPP σ-complex (F= 0.5 V/Å)

PBE/ all-electron numeric atom-centered basis, light computational settings

Total energy of the DFT calculation: -1109013.986855827 eV

Atom	x	y	z
C	-0.79523223	-3.46547396	1.58673529
C	-1.99588487	-2.73030056	1.55298386
C	-2.79758453	-2.72454601	0.41650534
C	-2.43787885	-3.46149735	-0.72531131
C	-1.39305860	-4.40001885	-0.58843610
C	-0.57418252	-4.38698227	0.53959440
C	-3.98490154	-1.96890118	-2.09951792
C	-3.82999365	-0.73042085	-2.69499758
C	-2.59770433	-0.35277558	-3.29694887
C	-1.68690434	-1.39763357	-3.58272797
C	-1.81717406	-2.64068225	-2.98410294
C	-2.89420597	-2.92431566	-2.05808457

C	2.36329505	-1.68444451	2.39753068
C	1.96076928	-0.87495741	3.48428388
C	0.83262485	-1.32186770	4.21128797
C	0.01170048	-2.33336336	3.71704564
C	0.27482336	-2.94069693	2.46691634
C	1.54624813	-2.69776485	1.90612595
C	2.41268950	0.53520674	3.49932588
C	3.59467151	0.97453035	2.85231939
C	3.64100384	2.20025581	2.18944606
C	2.50037917	3.04548494	2.12673892
C	1.49195063	2.76282390	3.08345210
C	1.44898323	1.54289920	3.74723576
C	0.94462013	4.57373548	0.83339271
C	2.12601615	3.78807461	0.90810726
C	2.62093522	3.32439618	-0.33953298
C	1.83718256	3.34245485	-1.48487694
C	0.49897020	3.81252959	-1.45431934
C	0.15866611	4.59127752	-0.31288900
C	-0.93895366	1.44265292	-3.97852589
C	-0.16003185	2.49155200	-3.51291039
C	-0.50530789	3.20409475	-2.33463788
C	-1.80897319	2.97129205	-1.83003446
C	-2.58769836	1.92172604	-2.29381434
C	-2.10626210	1.02849174	-3.28258590
H	-2.22219127	-2.02661799	2.35521520
H	-3.63051155	-2.02414832	0.36601506
H	-1.16964219	-5.09103914	-1.40287002
H	0.28007209	-5.06558747	0.57885342
H	-4.90350013	-2.18859849	-1.55290228

H	-4.65494826	-0.01871052	-2.64193625
H	-0.77270368	-1.19334397	-4.13976313
H	-0.98490845	-3.34156488	-3.05078798
H	3.23393630	-1.39697197	1.80587795
H	0.53150772	-0.80406775	5.12563365
H	-0.90259358	-2.59020004	4.25499990
H	1.81297906	-3.17860657	0.96398280
H	4.43679925	0.28675250	2.73985497
H	4.51819104	2.42495310	1.57660140
H	0.60194106	3.39275834	3.12671255
H	0.53346812	1.28390184	4.28163810
H	0.56505046	5.07473763	1.72870083
H	3.54492560	2.74443816	-0.36942573
H	2.19114063	2.79222898	-2.35745582
H	-0.79819763	5.11642079	-0.28565142
H	-0.60513769	0.88614463	-4.85506936
H	0.77055390	2.71974831	-4.03694087
H	-2.15314988	3.51143737	-0.94697609
H	-3.51265984	1.68754962	-1.76691716
Au	-3.82832896	-4.32749389	-3.40428808
Au	-5.21162369	-6.63434626	-4.44391517

4. Supplementary References

1 Cheng, Z. L. *et al.* In situ formation of highly conducting covalent Au-C contacts for single-molecule junctions. *Nat. Nanotechnol.* **6**, 353-357 (2011).

2 P. W. Atkins., J.C. de Paula. *PHYSICAL CHEMISTRY 10th Edition* (Oxford Univ. Press, Oxford, 2014)
